# The researchers’ role in knowledge translation: a realist evaluation of the development and implementation of diagnostic pathways for cancer in two United Kingdom localities

**DOI:** 10.1186/s12961-017-0267-8

**Published:** 2017-12-13

**Authors:** Jon Banks, Lesley Wye, Nicola Hall, James Rooney, Fiona M. Walter, Willie Hamilton, Ardiana Gjini, Greg Rubin

**Affiliations:** 1 National Institute for Health Research Collaboration for Leadership in Applied Health Research and Care West (NIHR CLARHC West), University Hospitals Bristol NHS Trust, Bristol, UK; 2 Centre for Academic Primary Care, Population Health Sciences, Bristol Medical School, University of Bristol, Bristol, UK; 3 University of Sunderland, Faculty of Health Sciences and Wellbeing, Sunderland, United Kingdom; 4 NHS South, Central and West Commissioning Support Unit, Bristol, UK; 5 Department of Public Health and Primary Care, Primary Care Unit, University of Cambridge, Cambridge, UK; 6 University of Exeter, Medical School, Exeter, UK; 7 Public Health England/NHS England, Chippenham, UK; 8 Wolfson Research Institute, Durham University, Durham, UK

**Keywords:** Knowledge translation, Realist evaluation, Qualitative research, Diagnostic pathways for cancer

## Abstract

**Background:**

In examining an initiative to develop and implement new cancer diagnostic pathways in two English localities, this paper evaluates ‘what works’ and examines the role of researchers in facilitating knowledge translation amongst teams of local clinicians and policy-makers.

**Methods:**

Using realist evaluation with a mixed methods case study approach, we conducted documentary analysis of meeting minutes and pathway iterations to map pathway development. We interviewed 14 participants to identify the contexts, mechanisms and outcomes (CMOs) that led to successful pathway development and implementation. Interviews were analysed thematically and four CMO configurations were developed.

**Results:**

One site produced three fully implemented pathways, while the other produced two that were partly implemented. In explaining the differences, we found that a respected, independent, well-connected leader modelling partnership working and who facilitates a local, stable group that agree about the legitimacy of the data and project (context) can empower local teams to become sufficiently autonomous (mechanism) to develop and implement research-based pathways (outcome). Although both teams designed relevant, research-based cancer pathways, in the site where the pathways were successfully implemented the research team merely assisted, while, in the other, the research team drove the initiative.

**Conclusion:**

Based on our study findings, local stakeholders can apply local and research knowledge to develop and implement research-based pathways. However, success will depend on how academics empower local teams to create autonomy. Crucially, after re-packaging and translating research for local circumstances, identifying fertile environments with the right elements for implementation and developing collaborative relationships with local leaders, academics must step back.

**Electronic supplementary material:**

The online version of this article (doi:10.1186/s12961-017-0267-8) contains supplementary material, which is available to authorized users.

## Background

This paper presents a realist evaluation of an initiative to develop and implement cancer referral pathways from primary to secondary care in two English localities.

Referring patients for cancer investigation is a challenging process; many people present to primary care with symptoms that may suggest cancer but are more frequently self-limiting and benign [1]. The difficulty for general practitioners (GPs) is to identify patients who are most likely to have cancer and refer them for fast-track investigation, which in the United Kingdom is colloquially termed the ‘2-week wait pathway’. In recent years, there has been an expansion of research and policy initiatives around cancer diagnostics in primary care [2–5].

The knowledge translation initiative examined in this paper sought to develop and implement referral pathways for cancer by combining different sources of knowledge. Knowledge translation is defined as, “*a dynamic and interactive process that includes the synthesis, dissemination, exchange and ethically sound application of knowledge to improve health, provide more effective health products and strengthen the healthcare system*” [6].

The initiative was led by applied health researchers working collaboratively as the ‘Discovery Programme’ [7] and included most of this paper’s authors (JB, NH, AG, FW, WH and GR). The aim was to draw on local knowledge and the research findings of the Discovery programme and other cancer diagnostic studies to create research-based pathways for implementation into local healthcare practice in two English localities. The intended role of the researchers was to furnish research to reference groups made up of local stakeholders who could then take the process forward. To learn more about what worked, we undertook a realist evaluation [8] and started by identifying the underlying programme theory to understand why combining academic research with local knowledge could be effective in the development of improved cancer pathways. Our initial programme theory was that drawing together up-to-date cancer diagnostics research (what works) and presenting it in an accessible format to a group of local stakeholders and policy-makers who have the authority to change pathways (context) will stimulate local ownership (mechanism) leading to the development of pathways incorporating academic knowledge into local settings. To evaluate this we employed the following research questions, (1) How do the people that make up the reference groups respond to academic research and how do they work with the Discovery research team who initiated the process? (2) To what extent do the new pathways draw on academic research? (3) How successful are the reference groups in getting the local pathways implemented? (4) What contextual factors help or hinder this process?

The initiative delivered very different results in terms of the design, completion and implementation of the pathways and subsequent outcomes in the two sites. Our hope was that a realist evaluation would identify the contextual factors and mechanisms that differentiated the two sites and enable guidance on how such initiatives might succeed in the future. The paper has been written in accordance with the RAMESES II reporting standards for realist evaluations [9].

### The initiative – combining academic research and local knowledge

The initiative was undertaken at the interface between primary and secondary care – GPs refer patients for investigation and secondary care teams undertake diagnostic investigations. There is a balance that has to be achieved between the two. As mentioned above, most people with cancer-like symptoms do not have cancer, and thus GPs face the task of evaluating the risk of non-referral (missing cancer cases) and too many referrals (high demand on secondary care investigative and diagnostic services). Cancer diagnostic research has sought to improve the balance between referral and investigation and several research-based tools have been developed, including risk assessment tools (RATs) and QCancer [5, 10]. The initiative to develop the pathways was an extension of this drive to improve cancer diagnostics in the United Kingdom, but the difficulty lay in negotiating the complex funding and policy relationships that exist between primary and secondary care. A further challenge was that, during the initiative, the organisations that manage 80% of National Health Service (NHS) funding were radically reorganised across England, which affected the sites involved [11].

The initiative was the final strand of the Discovery programme of research and was a pilot for attempting to put research into healthcare practice. Sites were identified based on local knowledge and links with Discovery team members. Local stakeholders were invited by members of the Discovery programme to form ‘reference groups’ in each locality to develop and implement diagnostic pathways for cancer. In site 1, Discovery member AG, who was also the regional cancer lead for public health, identified and invited reference group members via existing healthcare forums. In site 2, Discovery study member GR, an academic clinician, recruited a reference group chair who then jointly identified and recruited participants. Both reference groups included primary and secondary care clinicians, service managers, service providers and academics from the Discovery programme. Non-clinical local stakeholders included patient representatives and members of regional strategic clinical networks (organisations which combined clinical, service delivery and patient input to monitor and shape local healthcare provision). Permissions were gained from the local healthcare providers for an ‘in principle’ agreement to implement the new pathways in the two areas.

Pathway development was focused on three cancers in each locality, namely colorectal, lung and pancreas. These were chosen by the Discovery research programme team as the exemplar cancers of the programme because each contained different challenges in relation to diagnostic investigation [7].

A key element of the initiative was the introduction of academic knowledge into the reference groups. The Discovery team independently devised a customised information pack for each reference group consisting of a synthesis of cancer diagnostic information with up to-date research evidence, local cancer intelligence and resource materials to inform the process of pathway design. The documents were adapted for each site with locally pertinent data and contained:

1. An overview of the Discovery Programme and the plans for pathway development initiative.
2. An overview of the National Cancer Intelligence Network analysis of routes to cancer diagnosis in England and relevant, locality-specific data with a particular focus on the proportion of patients referred on the appropriate fast track pathways for cancer investigation.
3. An overview of primary care referral guidelines and diagnostic pathways for cancer at national and local levels.
4. Details of the resources developed by the Discovery research team with a particular focus on RATs [12–14] for the three cancers of interest. RATs are tools developed to help GPs select patients for cancer investigation by giving risk scores to particular symptoms or symptom combinations.

In addition, members of the Discovery team were on hand to verbally ‘translate’, when necessary.

## Methods

This study was approved by the University of Bristol, Faculty for Medicine and Dentistry, Committee for Ethics (FCE), Ref: 131448 (9402). Using a case study approach [15], the evaluation had two main phases.

Firstly, a documentary analysis of the meeting minutes and decisions taken by the two reference groups was carried out alongside mapping of the formation and shape of the pathways. This enabled a comprehensive understanding of the process and the overall outcomes in relation to pathway development and implementation. This process addressed the following questions:

1. Did the sites develop new pathways for each of the specified cancers?
2. Were the pathways developed within the study time frame?
3. To what extent did the sites draw on the research of the Discovery Programme and/or other recent cancer diagnostic research?
4. Were the developed cancer diagnostic pathways implemented?

JB, NH and JR met and examined documents associated with pathway development, including reference group minutes and the associated iterations of the pathway. This documentary analysis generated accounts of the pathway development in each locality, which fed into a comparative matrix table including data on both pathway content and timing (Table 1). The table also served to inform the development of draft context–mechanism–outcome (CMO) configurations, which, along with the programme theory, shaped our strategy for data collection in the second phase of the study.

**Table 1:** Comparing pathway modifications: changes and adaptions made to diagnostic pathways

Site 1	Site 2
Lung	
• Remove minimum time threshold for referral • Introduction of RATs as reference tool • For persistent high risk symptoms OR suspicious CXR patient referred simultaneously to 2-week wait pathway 2WW clinic AND CT scan 2WW	• Formal use of RATs alongside existing national NICE guidelines including a recommended risk assessment threshold of 2% • Option to do 2WW and simultaneous CXR for highly suspicious symptoms • Radiology given initiative to initiate 2WW referral and CT scan following suspicious CXR
Pancreas	
• RAT introduced as reference tool • Built on previous pilot by secondary care trust • Splits jaundice into a separate pathway (recognition of high risk) • Fast track for jaundice and suspicion of cancer • Simultaneous referral for CT and 2WW on non-jaundice pathway for high risk symptoms • Fast track route in for GP generated ultrasound referrals w/suspicion of malignancy	• Formal use of RAT and threshold score for 2WW • High risk symptoms go direct to CT scan followed by consultant review • Below national NICE guidance and RAT threshold consider abdominal US scan, if suspicious into 2WW
Colorectal	
• No change to national NICE guidelines	• Formal introduction of RAT with lower threshold than national NICE guidelines • For high risk symptoms and patients that meet the safety criteria GPs given a direct access to colonoscopy option

*2WW* 2-week wait referral pathway, *CT* computed tomography scan, *CXR* chest X-ray, *GP* general practitioner, *NICE* National Institute for Health and Care Excellence, *RAT* risk assessment tool, *US* ultrasound

This second phase used qualitative interviews with purposefully sampled participants from each reference group at the two sites, which enabled in-depth insight into the context and mechanisms at play, for example, the rationale behind the decisions that were taken in relation to pathway development. Participants were sampled purposefully to ensure representation from clinicians (primary and secondary care), service managers and non-clinical stakeholders. Participants were invited by e-mail, and interviews took place between December 2014 and February 2015, which was approximately 7 months (site 1) and 12 months (site 2) after the reference groups had completed the main aspects of pathway development. Interviews were undertaken by JR, who was not a member of the Discovery team, and broadly followed a topic guide but were not restricted to it (Additional file 1). The topic guide was developed from an assessment of the pathway development documentation described above and the programme theory that was outlined at the start of the evaluation. Formal consent to participate was taken prior to the interview.

Twenty members of the reference groups were invited via e-mail, of which 14 agreed to participate (Table 2) and 6 did not respond. Most interviews took place over the phone (n = 10) with the remainder face to face (n = 4). Interviews were audio recorded, transcribed verbatim and fully anonymised.

**Table 2:** Interviews

	Primary care	Secondary care	Non-clinical	Total
	Interviewed (invited)	Interviewed (invited)	Interviewed (invited)	Interviewed (invited)
Site 1	2 (3)	5 (7)	1 (1)	8 (11)
Site 2	3 (3)	1 (4)	2 (2)	6 (9)

Interview transcripts were analysed thematically [16]. Initially, transcripts were read by JB and JR, and an initial coding frame was developed. Two transcripts were double coded by JB and JR, and the codes were further refined. JR coded the full set of transcripts using NVivo version 10 [17]. Coded data were organised into CMO configurations (e.g. codes for ‘working relationships’ and ‘leadership’ were categorised as contextual factors). JB, JR and NH engaged in a process of testing and refining the programme theory and developing CMOs, at regular research meetings as data analysis continued, until the final CMOs and revised programme theory were agreed. These are presented below.

## Results

### Pathway development – overview of outcomes

Table 1 provides an overview of the differences in the pathway content in the two sites by cancer type. Pathways were developed in both sites for the diagnosis of lung and pancreas cancers. Both aimed to accelerate diagnosis by employing simultaneous referrals for tests and investigations alongside referrals to secondary care clinical teams. This would enable a patient to present for specialist assessment in secondary care with relevant tests completed. However, the reference group in site 1 did not reach agreement on a new pathway for suspected colorectal cancer. Thus, only two pathways were developed in site 1 rather than three, as in site 2.

A second major difference was the inclusion (or not) of RATs. In site 1, the reference group stopped short of formally incorporating RATs as part of the assessment for referral and instead included them as reference tools for guidance. However, in site 2, the reference group incorporated RATs from the Discovery research programme into all three pathways.

There was a difference in the time it took to develop the pathways. The pathways in site 1 took 3 months longer to be developed and also involved twice as many meetings (Table 3).

**Table 3:** Reference groups timing and launch

	Site 1	Site 2
Meeting 1		
Date of meeting	29/07/2013	01/09/2013
Meeting structure	3 sub-groups established for each cancer pathway Chaired by Discovery research lead, 1 PPI member	3 sub-groups established for each cancer pathway Chaired by local stakeholder 1 observer from National Cancer Action Team, 2 PPI members and 2 members of Discovery research team
Post-meeting actions/activity	Discovery research team collate minutes and design pathways	Local reference group members action the pathway design
Meeting 2		
Date of meeting	11/11/2013	13/11/2013
Meeting structure	No sub-groups – pathway changes reviewed by full reference group Chaired by Discovery research team lead Significant change in membership/attendance	No sub groups – pathway changes reviewed by full reference group Chaired by local stakeholder 2 Discovery researchers present
Meeting outcome	Pathways presented by Discovery research team not accepted by reference group and required further work	New pathways considered and accepted with minor amendments agreed prior to implementation
Post-meeting actions/activity	Discovery team liaise with clinical leads from reference group to redraw pathways	Local reference group members amend pathways
Meeting 3		
Date of meeting	21/01/2014	N/A – pathway design completed
Meeting structure	No sub-groups – pathway changes reviewed by full reference group Chaired by Discovery research team lead Significant change in membership	
Meeting outcome	Pathways submitted by Discovery team were not fully accepted by reference group; further work required	
Post-meeting actions/activity	Discovery team liaise with clinical leads from reference group to redraw pathways 2 meetings with colorectal sub-group failing to reach agreement	
Meeting 4		
Date of meeting	11/03/2014	N/A – pathway design completed
Meeting structure	Pathway changes reviewed by full reference group Chaired by Discovery researcher	
Meeting outcome	Lung and pancreas pathways subject to further amendment and tentatively agreed No agreement on colorectal pathway	
Launch and promotion of modified cancer pathways		
Launch meeting date	01/05/2014	14/02/2014
Meeting details	8 GPs in attendance No protected GP time, i.e. GPs attending in their own time Discovery team lead with support from clinical reference group members	~230 GPs attended Protected time for GPs as part of ‘time in time out’ training day Pathways presented by reference group team Short intro from Discovery researcher

*GP* general practitioner, *PPI* patient and public involvement representative, *N/A* not available

However, perhaps the most important difference between the two sites was that the pathways were not fully implemented in site 1. Technical difficulties were encountered loading the details on the electronic GP referral system and these were never fully integrated; the referral forms still gave standard national guidance and did not give the option for simultaneous referral, which was a key aspect of the newly designed pathways. In short, when site 1 GPs logged onto the system there was no clear way of using the new pathways, whereas in site 2 GPs were able to fully access the re-designed pathways.

Both sites held launch events to promote the pathways (Table 3), but there were differences in the role of the Discovery team. In site 2, the launch was organised by reference group members and drew on pre-existing event processes and organisational structures within the local healthcare system. Around 230 GPs attended as the launch was tied into a regular GP training session and GPs had protected time to attend. However, in site 1, the Discovery research team organised the launch. Although the research team advertised in a widely distributed electronic newsletter, they were not able to tap into the same local infrastructure and only eight GPs attended. In site 1, there was a stronger association of the new pathways with the research team, which did not carry the same level of ‘official’ (NHS) endorsement or backing.

### The CMO of pathway development

Data analysis from the two phases led to the production of four CMO configurations which identified the elements that contributed to successful pathway development and implementation, and explained the differences between the two sites.

#### CMO 1. A stable group made up of the ‘right’ people who have previously worked together successfully (context) facilitates a shared purpose (mechanism), which leads to effective and timely pathway development (outcome)

Different patterns of attendance emerged from the reference group meetings (Table 4). The site 1 group had very little compositional stability, whereas in site 2 there were few changes of personnel. Table 4 shows the change in proportion of roles that populated the two groups over the duration of pathway development. The site 2 group change was < 10% for all roles whereas in site 1 there was an increase in secondary care membership of 19% across the four meetings (this was as high as 61% at meeting 3). There was also a decrease in primary care membership of 22% in site 1.

**Table 4:** Reference group composition

	Site 1					Site 2		
Profession/role	Meeting 1	Meeting 2	Meeting 3	Meeting 4	Change (%)	Meeting 1	Meeting 2	Change (%)
Secondary care clinician	4 (31%)	6 (46%)	11 (61%)	6 (50%)	+19%	5 (28%)	3 (20%)	–8%
Secondary care manager	0 (0%)	2 (15%)	3 (17%)	1 (8%)	+8%	1 (6%)	1 (7%)	+1%
Primary care clinician	4 (31%)	2 (15%)	0 (0%)	1 (8%)	–22%	5 (28%)	5 (33%)	+6%
Research team	4 (31%)	3 (23%)	4 (22%)	3 (25%)	–6%	2 (11%)	2 (13%)	+2%
Clinical network	0 (0%)	0 (0%)	0 (0%)	0 (0%)	0%	3 (17%)	2 (13%)	–3%
Patient/public	1 (8%)	0 (0%)	0 (0%)	1 (8%)	+1%	2 (11%)	2 (13%)	+2%
Total	13	13	18	12		18	15	

The importance of group composition was highlighted by a site 2 member,

“*I think the benefit of it obviously was getting people in the same room at the same time so obviously having a mix of different people from primary care, from secondary care, from trust management, that obviously needs to happen if you’re going to try and make changes like the ones that have been made … I don’t think it would have worked so well if you had – if you didn’t have the right people in the room.*” (Site 2, 004)

The perception of a well-balanced reference group in site 2 was underpinned by a legacy of participants having previously worked together on change and implementation initiatives. There was a shared network and history to build on which conveyed a feeling of continuity between the Discovery programme initiative and previous projects. There was also a legacy of developing policy change in site 2, which conveyed a sense of continuity between the Discovery work and previous change initiatives,

“*I think the personal relationships between the participants were quite important, that these were people who’ve worked together for some time and who have interacted through the cancer network previously and I think that helped.*” (Site 2, 003)

The strong group identity at site 2 appeared to override the members’ individual agendas and organisational interests,

“*I’ve always been struck by the collaborative way in which clinicians come together on cancer services. … they genuinely behave as if it’s a service that’s important not their organisation. We do talk about people taking their team’s shirt off as they come in the door and they do, you know, in these workshops that we’ve organised over the years people are very keen to talk about the service and patients not the organisational politics but it inevitably on some occasions does come into play.*” (Site 2, 003)

In contrast, there were several negative reflections on the composition of the site 1 group. A common perception was that key people were missing, but there was no consensus on who should have been present. For some, it was the absence of secondary care clinicians and for others it was a lack of primary care clinicians. Moreover, while some members in site 1 had previously worked together, the group as a whole had not and the constant fluidity of membership made it difficult to establish a shared purpose.

#### CMO 2. Respected, independent and well-connected local leaders who model partnership working across organisational boundaries (context) foster engagement from reference group members (mechanism) who are proactive in pathway design and development

In site 2, there was evidence of respected and trusted leaders within the reference group. More than one person led the process in site 2. Clinical leadership came from a GP with established cancer-related interests with several organisational bodies in the region,

“*I guess the lead GP, if we could describe as such, would be XX … he’s very committed, he’s a very committed GP to cancer and I think he is now, I think at that point he was becoming an executive – he was becoming part of the executive of the* [policy-making organisation] *and he was leading on cancer so that was extremely helpful.*” (Site 2, 003)

Further clinical leadership came from a clinician with a high profile and a previous leadership role within the cancer networks,

“*We are lucky enough as well, we always mention about YY. He used to be the cancer network director but he’s still running the clinical – he’s actually running the clinical network now so cancer’s one of it and he is very good at bringing people together … YY actually helped us – helped myself and some of the key people learn how to work together and now when you see that come along we actually knew how to do it and we could take it forward. YY is absolutely crucial.*” (Site 2, 006)

Having successfully worked across boundaries, particularly primary and secondary care, these leaders provided valuable learning on partnership working, which was beneficial to the site 2 reference group, who were also drawn from different healthcare sectors and clinical specialities. These leaders helped site 2 reference group members know ‘how to do it’ and fostered proactive engagement. This proactivity was evidenced in the documentary analysis (presented in Table 3), with site 2 reference group members taking most of the responsibility for the work between meetings, while site 1 reference group relied on significant input from the Discovery research team, including taking forward the actions of previous meetings.

In site 1, leadership was compromised for several reasons. The chair of the reference group was also part of the Discovery research team, so the site 1 group did not benefit from the perception of independent leadership present in site 2. Additionally, a major re-organisation of the health service [18], which was contemporaneous with the study, led to the chair changing her organisational base and a destabilisation of institutional ties and connections. This resulted in a perceived lack of continuity with key sectors of the local healthcare economy, partnership working was frustrated and the new association with different institutions further compromised the perception of independence. Consequently, engagement was poorer.

#### CMO 3. A clear understanding and acceptance of the aims of the project, including the legitimacy of research data and the process of pathway development (context), provides a basis for agreement (mechanism), which facilitates a pathway incorporating research evidence (outcome)

In site 2, there was a clearer understanding, acceptance and consensus of why the work needed to be undertaken,

“*Yes, we were aware that late diagnosis was a significant problem; in fact it still is across the entire health service and in our area in particular. We could see that there were areas doing better than us and this was reflected in mortality statistics we were getting from public health.*” (Site 2, 009)

In site 1, respondents agreed with and supported the aims of improving cancer diagnosis and believed improvements were possible, but some thought the project was focusing on the wrong part of the pathway. The overall initiative, its legitimacy and aims were brought into question much more in site 1.

“*There was some concerns as to how realistic the project was in its overall aims, could we actually make a difference in such a short time and then measure it and I think we all had concerns about that … there was an aim to get it rolled out across the whole of* [site 1] *how realistic was that to influence how GPs practice, ‘cos we know it’s very difficult to change people’s practice and to change everyone in* [site 1] *to suddenly changing pathway was quite an ask, and how robust therefore the data be so I think we were a bit concerned that the methodology, whether it was actually robust*.” (Site 1, 008)

This contributed to a negative or defensive attitude to the initiative,

“*In terms of, you know, spending anyone’s money on the lung cancer part of this I thought that probably wasn’t the most prudent way to do it and I’d much rather been given a chunk of money and been told to go and do some social marketing to try and influence things … I think we needed to shape it because it would have been even worse, wouldn’t it, if none of us had turned up*.” (Site 1, 013)

The differing perspectives on the initiative and the Discovery team were also reflected in the way that the local reference document was viewed in each site. As described above, the research team compiled the documents prior to the first meeting in both sites. Site 2 viewed the reference document as a platform to start the process of pathway design; they recognised the contents as valid and relevant, whereas in site 1 the contents of the documentation and research data were contested and their relevance to the local area questioned,

“*There’s public health involvement which to my mind was quoting figures that I didn’t recognise … I think you have to use very well validated data to show what the problem is … I know that our data is very robust and I can tell you exactly how many cases of lung cancer I saw last year presented as an emergency and it’s not many*.” (Site 1, 013)

#### CMO 4. The research team take a minor, non-directive role in the reference group (context), which encourages local ownership (mechanism) and leads to proactive pathway design and support for implementation (outcome)

The Discovery programme research team adopted different approaches to their relationship with the reference groups and their role in developing the pathways. In both sites, the research team compiled, distributed and ‘translated’ the information packs after identifying local leaders and stakeholders to take the initiative in developing and designing the pathways. Then, in site 2, the research team stepped back from the process. However, in site 1, the research team had a major role in every meeting as a Discovery team member chaired the meetings and other Discovery researchers regularly attended and were actively involved in pathway design (Table 3). Crucially, as the process faltered in site 1 so the research team tried to rescue the initiative by becoming more prominent in shaping the pathways. In site 2, the research team were much more in the background.

Consequently, the process was viewed very differently in the two sites. In site 1, it was perceived as a top-down process led by the research team who were trying to ‘impose’ a set of ideas on the local healthcare system,

“*It felt very much like we were being told you have to do this rather than here’s a project, here’s our aims, we’d like to do this, can you support us and help us, you know, let’s do this together. I think there perhaps wasn’t so much of a collaborative kind of feeling in the way it was done. It was always a bit vague.*” (Site 1, 002)

Conversely, in site 2, the ‘light touch’ approach taken by the research team led to a feeling of ownership by the reference group, which gave momentum and energy. The research team remained external yet supportive to the reference group so that when the revised pathways were fed back at subsequent meetings, the pathways were recognised as belonging to the reference group, rather than a product of the Discovery team researchers.

## Discussion

### Summary of results

**Fig. 1:**
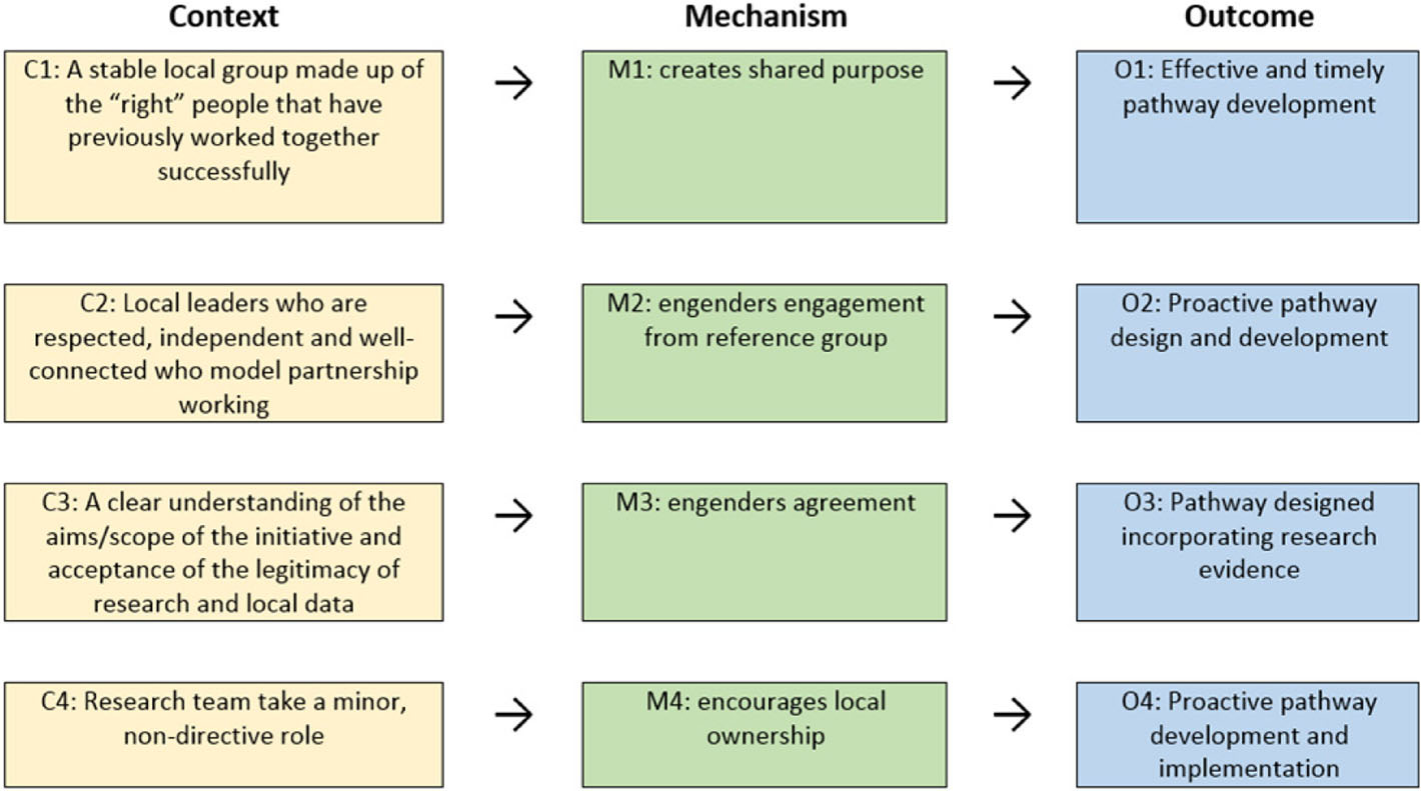
Context–mechanism–outcome configurations

We identified four CMOs that were instrumental in combining academic research with local knowledge to feed into the development and implementation of new pathways for cancer diagnosis. A key finding was that success was greater when pathway development and implementation was performed by local stakeholders who were more autonomous from the research team. Figure 1 sets out these CMO configurations.

Our original programme theory suggested that combining academic and local knowledge would foster local ownership leading to the successful development and implementation of research-based pathways. However, on completion of data collection and analysis, we recognised that the furnishing of knowledge alone was insufficient and that engendering ‘ownership’ was more nuanced. The empowerment and autonomy of the local teams were essential to the success of the initiative. Thus, our revised programme theory is that a respected, independent, well-connected leader who models partnership working and facilitates a local, stable group with a strong identity that agrees about the legitimacy of the project aims, process and data, working with a research team who take a minor, non-directive role (context) can empower local teams to become sufficiently autonomous (mechanism) to develop and implement research-based pathways (outcome).

The more successful site had a balanced, stable membership with a history of working together, which created a strong sense of identity and fostered a greater sense of shared purpose – a mechanism that helped drive the process. Moreover, site 2 benefited from respected, well-connected and independent local leaders with excellent collaboration skills who had consistent inter-organisational ties for the duration of the project. This modelling of partnership-working empowered site 2 reference group members to gain the confidence and skills necessary to make the project a success. Possibly because these leaders were trusted, this helped the reference group to understand and accept the legitimacy of the information pack in a way that the research team in site 1 could not. Moreover, site 2 leads had strong roots in the local clinical networks and brought an independence to the group, i.e. they were not seen representing any organisational interests other than the improvement of cancer services. Their autonomy gave the process momentum that was lacking in the other site, and more work was completed between meetings without input from the research team.

Site 1 provided a stark contrast. With less stable membership, which did not have an established history of working together, a weak sense of identity and changing local structures, the pathway development process became almost a defence against the perceived imposition of new pathways by the research team, as the researchers attempted to compensate for key missing elements. The research team were not seen as independent or credible and dialogue was more adversarial. The reference group questioned the need for reform at ‘their’ point of the pathway and were sceptical of the research materials that were used to start the process; there was not the shared understanding that existed in site 2. Further, political changes with re-organisations had a much more disruptive effect at site 1, where leadership positions were affected. Site 1 reference group members felt neither empowered nor autonomous. This does not mean that knowledge translation should be avoided in more complex localities during unstable times, but it is an indication of where it might be easier.

### Implications of findings

This paper raises important questions about researchers’ roles in dissemination and implementation. The Discovery research team consisted of applied health services researchers with little expertise in knowledge translation. Increasingly, academics will also need to acquire knowledge translation skills as funding bodies are placing greater emphasis on viable dissemination plans and core university funding relies on demonstrations of ‘impact’ within the United Kingdom [18, 19]. Just as all health services academics have had to address issues of patient engagement with their research, so they will need skills in knowledge translation; it is becoming everybody’s business.

Consequently, researchers are being asked to adopt new roles. Mode 1 is the default with the researcher as a ‘reflective scientist’, but there is the emerging role of Mode 2, with the researcher as an ‘intermediary’ or ‘facilitator’ [20]. For example, there is an account of Mode 1 Norwegian researchers acting in Mode 2 roles to set up a randomised controlled trial in cooperation with policy-makers, only for the researchers to become frustrated as the policy-makers implemented the less effective intervention [21]. To help educate and up-skill researchers, more studies are needed, like this one, whereby researchers experiment and evaluate how they can operate effectively within the world of Mode 2 without prejudicing the quality of their science.

The intention of the Discovery researchers was to be responsible for the synthesis and presentation of research and local data, and leave local stakeholders in charge of pathway development and implementation. The research team hoped to identify and cultivate collaboration with an enthusiastic local leadership, who would convene the reference group and drive the implementation forward. Then, the Discovery team intended to step back. This approach worked successfully in site 2, where the key elements of successful pathway development and implementation were in place, so that reference group members felt sufficiently empowered and autonomous. The researchers attempted to use the same approach in site 1, but with two key differences – the key local contextual elements were not in place and the research team took on a leadership and active design role, which failed to work.

The key learning point was that researchers cannot compensate for the missing elements of successful knowledge translation. If those elements do not exist, given the challenges of implementation, research teams may need to move on to more fertile ground. Local stakeholders, not researchers, have to undertake implementation. Similarly, researchers need to be aware that initial enthusiasm may ebb. However, if in an effort to save the project, research teams attempt a rescue operation, they are unlikely to achieve their overall goal – that of seeing the results of their research implemented.

### Strengths and limitations

A major strength of this research was that two sites were studied, such that potential CMOs could be contrasted and compared to create more robust theories about what worked. However, these CMOs need further testing elsewhere to provide greater confidence in the generalisability of findings.

An unusual aspect was that the main interviewer was a policy-maker. With his experience of managing change in healthcare economies, he brought extra depth to the data collection, analysis and interpretation process.

A key challenge of this study was the continual changes in healthcare policy and arrangements nationally and locally that required the pragmatic adaptation of the research team.

## Conclusion

Our study adds useful information to the literature about the role that applied health services researchers can adopt to encourage implementation of research findings, and the contextual factors that are more likely to lead to local uptake. We found that, despite a comparable ‘input’ of locally tailored research evidence, outcomes for the two sites were different. Although both local stakeholder groups designed relevant, evidence-based cancer pathways, the research team assisted in the site where the pathways were successfully implemented, while in the other the research team drove the initiative. Consequently, the local teams were not empowered or autonomous, which meant that the resulting pathways were never ‘owned’ locally nor implemented. To facilitate successful implementation, research teams need to re-package the scientific evidence for local circumstances, ‘translate’ that evidence verbally, identify fertile environments with the right elements for implementation, develop a collaborative relationship with a local leader(s) to take action and, then, importantly, step back.

## Additional file

Additional file 1: Interview schedule/topic guide. (DOCX 15 kb)

## Abbreviations

CMO: context, mechanism, outcome
GP: general practitioner
NICE: National Institute for Health and Care excellence (UK)
RAT: risk assessment tool
