# Supplementary material for: The researchers’ role in knowledge translation: a realist evaluation of the development and implementation of diagnostic pathways for cancer in two United Kingdom localities
Source: Health Res Policy Syst. 2017 Dec 13;15:103. doi: 10.1186/s12961-017-0267-8 (PMC5729249; doi:10.1186/s12961-017-0267-8)
Supplement: Additional file 1: — Interview schedule/topic guide. (DOCX 15 kb) [file 12961_2017_267_MOESM1_ESM.docx]

**Additional file 1 – Interview schedule/topic guide**

**CDAPT Interview topic guide v3 28102014**

##### Background

- Tell us how you came to be involved in the CDAPT pathways project
  - Tell us about your work and how this related to the CDAPT project?
  - What was your role within the reference group?
- Did you perceive a need for change prior to the CDAPT pathways?
- Had there been changes in the cancer pathways prior to the CDAPT process

##### The Process - Pathway development and the Reference group

- Reflecting on the development of the pathways and the use of the reference groups – tell us what you thought of the process
  - How well did it work
  - What factors **facilitated** and what factors **inhibited** pathway development
  - Were there other changes – nationally or locally - that impacted on development (NHS/trust/cancer networks), national or local
  - Were their issues related to this area that affected the development of the pathway
  - Did costs / resources impact on the development of the pathway

##### The reference group

- What do you think of the make up of the reference group – was it balanced in terms of …
  - The 3 cancer sites
  - Primary and secondary care
  - Clinical, non-clinical and academic
- Were you involved in communication within the reference group between meetings
- How did the process – the use of the CDAPT reference group - compare with other examples of change in your organisation?
- Do you feel there was sufficient patient involvement in the process of pathway development

##### Pathways into practice

- What impact do you think the pathway has had / will have on clinical roles
  - E.g. clinical responsibility - do GPs have more clinical responsibility within the pathway (could also apply to radiologists and nurses)?
  - Has clinical responsibility changed with the introduction of referral tools such as the RAT
- Could you comment on the implementation process?
  - How were the pathways introduced locally
  - Have there been any problems
    - In introducing the pathways
    - In using the pathways
- Do you feel that the pathways have the support and resources to work (‘support’ at a clinical, CCG or secondary care trust level)

##### Concluding/summary questions

- What would you have done differently in the pathway development process
- Do you think the process was worthwhile … do you think the pathways will improve the referral process
